# CD73 mediates the therapeutic effects of endometrial regenerative cells in concanavalin A-induced hepatitis by regulating CD4^+^ T cells

**DOI:** 10.1186/s13287-023-03505-2

**Published:** 2023-09-29

**Authors:** Hong Qin, Chenglu Sun, Dejun Kong, Yanglin Zhu, Bo Shao, Shaohua Ren, Hongda Wang, Jingyi Zhang, Yini Xu, Hao Wang

**Affiliations:** 1 Department of General Surgery, Tianjin Medical University General Hospital, 154 Anshan Road, Heping District, Tianjin, 300052 China; 2 Tianjin General Surgery Institute, Tianjin Medical University General Hospital, Tianjin, 300052 China; 3 School of Medicine, Nankai University, Tianjin, 300071 China

**Keywords:** CD73, Endometrial regenerative cells, Concanavalin A-induced hepatitis, CD4^+^ T cell, Mice

## Abstract

**Background:**

As a kind of mesenchymal-like stromal cells, endometrial regenerative cells (ERCs) have been demonstrated effective in the treatment of Concanavalin A (Con A)-induced hepatitis. However, the therapeutic mechanism of ERCs is not fully understood. Ecto-5`-nucleotidase (CD73), an enzyme that could convert immune-stimulative adenosine monophosphate (AMP) to immune-suppressive adenosine (ADO), was identified highly expressed on ERCs. The present study was conducted to investigate whether the expression of CD73 on ERCs is critical for its therapeutic effects in Con A-induced hepatitis.

**Methods:**

ERCs knocking out CD73 were generated with lentivirus-mediated CRISPR-Cas9 technology and identified by flow cytometry, western blot and AMPase activity assay. CD73-mediated immunomodulatory effects of ERCs were investigated by CD4^+^ T cell co-culture assay in vitro*.* Besides, Con A-induced hepatitis mice were randomly assigned to the phosphate-buffered saline treated (untreated), ERC-treated, negative lentiviral control ERC (NC-ERC)-treated, and CD73-knockout-ERC (CD73-KO-ERC)-treated groups, and used to assess the CD73-mediated therapeutic efficiency of ERCs. Hepatic histopathological analysis, serum transaminase concentrations, and the proportion of CD4^+^ T cell subsets in the liver and spleen were performed to assess the progression degree of hepatitis.

**Results:**

Expression of CD73 on ERCs could effectively metabolize AMP to ADO, thereby inhibiting the activation and function of conventional CD4^+^ T cells was identified in vitro*.* In addition, ERCs could markedly reduce levels of serum and liver transaminase and attenuate liver damage, while the deletion of CD73 on ERCs dampens these effects. Furthermore, ERC-based treatment achieved less infiltration of CD4^+^ T and Th1 cells in the liver and reduced the population of systemic Th1 and Th17 cells and the levels of pro-inflammatory cytokines such as IFN-γ and TNF-α, while promoting the generation of Tregs in the liver and spleen, while deletion of CD73 on ERCs significantly impaired their immunomodulatory effects locally and systemically.

**Conclusion:**

Taken together, it is concluded that CD73 is critical for the therapeutic efficiency of ERCs in the treatment of Con A-induced hepatitis.

**Supplementary Information:**

The online version contains supplementary material available at 10.1186/s13287-023-03505-2.

## Introduction

Autoimmune hepatitis (AIH) is a liver disease caused by T cell-mediated autoimmune response in individuals who are genetically susceptible [1]. Acute episodes of AIH can lead to acute liver injury, even liver failure or death [2]. Though the pathophysiological mechanisms of acute liver injury in AIH are incompletely understood, the sharp release of adenosine triphosphate (ATP) and the excessive response of CD4^+^ T cells against liver cells has been identified to play a significant role during the pathogenies [1, 3, 4].

Extracellular accumulation of ATP plays a pivotal role in the initiation of immunogenesis [5]. Under physiological conditions, the concentrations of ATP are very low, which would be dramatically elevated under cellular stress conditions, such as inflammation, ischemia and hypoxia, due to ATP from intracellular storage pools into the extracellular compartment [6, 7]. The elevation of ATP concentration in the extracellular space and subsequent activation of purinergic type 2 receptors (P2R) on immune cells are considered as a major signaling pathway involved in inflammatory responses [6]. In a murine acute hepatitis model, large amounts of ATP were released from livers and then induced expression of P2Y2R on immune cells, which greatly promoted liver damage and necrosis [8]. In the metabolism of proinflammatory signal ATP, CD39 converts ATP to adenosine monophosphate (AMP). AMP is further hydrolyzed by Ecto-5`-nucleotidase (CD73) to adenosine (ADO), which can activate purinergic type 1 receptors (P1R) on immune cells and performs the opposite effects of ATP and AMP in immunomodulation [9]. As a key enzyme in the metabolism of AMP to ADO, CD73 regulates the balance of purinergic metabolism, which drives a shift from an ATP-induced pro-inflammatory environment to an ADO-driven anti-inflammatory milieu and may dictate the outcome of several pathophysiological events, including autoimmune diseases [10, 11].

Endometrial regenerative cells (ERCs), a kind of mesenchymal-like stromal cells derived from adult menstrual blood, exhibit the same immunomodulatory ability as mesenchymal stem/stromal cells (MSCs) and possess many advantages than MSCs [12, 13]. We and others previously have demonstrated ERC’s valuable effectiveness in treating autoimmune and inflammatory diseases, including experimental hepatitis by the inhibition of CD4^+^ conventional T cells [14–16]. Currently, studies on the immunomodulatory mechanisms of ERCs are relatively rare in comparison with MSCs. Some cytokines such as PG-E2, PD-L1, indoleamine 2,3 dioxygenase (IDO) and IL-10 have been demonstrated vital in the immunomodulatory effects of ERCs [17, 18]. As a type of mesenchymal-like stromal cells, ERCs widely express the surface marker CD73 which plays a key role in the regulation of purine metabolic pathway [10, 12]. However, whether CD73 is critical for the immunomodulatory abilities of ERCs in acute hepatitis has rarely been studied.

Concanavalin A (Con A)-induced hepatitis is a well-established murine model of T cell-mediated acute hepatitis [19]. In this study, we utilized Con A-induced hepatitis model to identify the significance of CD73 expression on ERCs’ immunomodulatory abilities and explore the potential mechanisms.

## Materials and methods

### Isolation, expansion, and gene modification of ERCs

ERCs were isolated from adult menstrual blood collected from volunteers as described previously [12]. Briefly, mononuclear cells were isolated by Ficoll density centrifugation from collected menstrual blood. The procedure was under ethical approval from Tianjin Medical University General Hospital (Tianjin, China, IRB2022-WZ-081). The isolated cells were then cultured in Dulbecco’s Modified Eagle’s Medium/Hams F-12 Mix medium (DMEM/F12, CORNING, USA) supplemented with 10% Fetal Bovine Serum (FBS, CORNING, New Zealand), and 1% penicillin/streptomycin (Solarbio, Beijing, China). The culture medium was changed every two or three days to remove the non-adherent cells and tissue fragments. The adherent cells were passaged with 0.25% Trypsin (with EDTA; Procell, Wuhan, China) and passage-3 cells were used for the identification of ERCs.

The *NT5E* gene (code CD73) deletion in ERCs was generated with lentivirus-mediated CRISPR-Cas9 knock-out technology (GeneChem, Shanghai, China). SgRNA sequences targeting human *NT5E* were designed as follows: 1^#^, CACCGAACTCATCGCTCAGAAAGT; 2^#^, CACTTTCTGAGCGATGAGTT. The lentivirus transfection procedure was done in a biosafety cabinet with an appropriate multiplicity of infection (MOI = 30). The transfected cells were passaged at 80% confluence. After selection with 2 μg/mL puromycin (Solarbio, Beijing, China), the efficiency of gene knockout was examined and subsequent experiments were performed.

### Western blotting

The cell total protein extraction and western blotting were performed as previously described [14]. To be specific, the total protein was extracted from cells by RIPA buffer (Solarbio, Beijing, China) supplemented with Protease Inhibitor Cocktail (Bimake, USA). Protein samples were mixed with 4 × loading buffer (Solarbio, Beijing, China) and boiled for 5 min at 95 °C. Next, 30 μg of total protein was separated with 10% SDS-PAGE gel. After blocked with 5% nonfat milk, membranes were incubated overnight at 4 ℃ with the anti-CD73 (clone EPR6114, dilution at 1:1000, Abcam, USA) antibodies diluted in Primary Antibody Dilution Buffer (Solarbio, Beijing, China). Then, they were washed with TBST, and the membranes were incubated for 60 min with horseradish peroxidase-linked goat-anti-rabbit secondary antibody (dilution at 1:2000, Cell Signaling Technology, USA). Finally, membranes were developed with the electro-chemiluminescence solution (Millipore, USA), and images were taken on a ChemiScope exposure machine (Clinx Science Instruments Co, Ltd, Shanghai, China).

### Analysis of AMPase activity by phosphate assay

The AMPase activity of ERCs was analyzed with the help of the Phosphate Assay Kit- PiColorLock (Abcam, USA). To be specific, on the first day, ERCs, NC-ERCs, and CD73-KO-ERCs were harvested and transferred to 96-well plates for 5 × 10^4^ cells per well. The next day, the culture medium was discarded and the plates were washed three times with saline, then the Phosphate Assay Kit was used to detect the AMPase activity of these cells. In short, 200 μL of AMP (100 μM, MedChemExpress, Shanghai, China)-containing saline was added to each well and incubated at the temperature of 37 ℃ for 10, 30, 60, 120, and 180 min, respectively. Then, 50 μL of PiColorLock reagent mix (PiColorLock reagent and 1/100 volume of Accelerator) was added into the Pi-containing culture supernatant. Next, 5 min later, add 20 µL of the Stabilizer, then mix with the pipette. Finally, after 30 min of incubation, the plates were counted absorbance at 635 nm via the Microplate Reader (TECAN, Switzerland).

### Preparation and co-culture of mouse CD4^+^ T cells

Mouse CD4^+^ T cells were isolated from the spleen by positive sorting using the CD4 (L3T4) MicroBeads (Miltenyi Biotec, Germany). The purity of the isolated cells was assessed by flow cytometry (shown in Additional file 1: Figure S1, purity of CD4^+^  > 95%). Purified CD4^+^ T cells were cultured in RPMI-1640 medium (CORNING, USA) containing 10% FBS (CORNING, New Zealand) and 50 IU/mL recombinant IL-2 (Peprotech, USA) with 5 μg/mL pre-coated anti-mouse CD3 (17A2, Biolegend) and 3 μg/mL anti-mouse CD28 (37.51, BioLegend).

In co-culture assay, ERCs, NC-ERCs, and CD73-KO-ERCs were seeded, respectively, at a density of 5 × 10^4^ cells/well and CD4^+^ T cells were seeded at 25 × 10^4^ cells/well in 24 well plates. When indicated, AMP (50 µM, MedChemExpress, Shanghai, China) was added. At the time of harvest (72 h), the cell membrane expression of the activation markers CD69 and CD154, and the dilution of CFSE as a measure of proliferation were assessed by flow cytometry. Besides that, the population of CD4^+^IFN-γ^+^ cells were detected by flow cytometry, and the IFN-γ levels in cell culture supernatants were determined by ELISA (DAKEWE, Beijing, China).

### Animals and experimental groups

C57BL/6 mice, 6–8 weeks old and 22–24 g weight, were used for the induction of Con A-induced hepatitis. All animals were housed in a conventional experimental environment with enough food and water following the guidelines of the China Association for the Production of Animals, and the animal experiment protocol was approved by the Animal Ethical and Welfare Committee of Tianjin Medical University General Hospital (Ethic No. IRB2022-DW-21).

Mice were given a single injection of Con A (Solarbio, Beijing, China) via the tail veins at 15 mg/kg of body weight dissolved in 200 μL of phosphate-buffered saline (PBS). For the main cell transplantation experiment, model mice were randomly assigned to the following four groups (*n* = 6 per group): untreated group; ERC-treated group (naïve ERCs); NC-ERC-treated group (ERCs transfected with lentiviral lacking *NT5E* gene, used as negative control); and CD73-KO-ERC-treated group (ERCs transfected with lentiviral to delete *NT5E* gene). As for treatments, 1 × 10^6^ cells per mouse were administered intravenously 30 min after the injection of Con A in ERC-, NC-ERC-, and CD73-KO-ERC-treated groups. 24 h after administration of Con A, all mice were euthanized with CO_2_ (at a rate of 40% replacement/min in a transparent 2 L chamber for at least 10 min). Serum, liver, and spleen samples were collected and processed for further analysis.

### Cell tracking in vivo

To track the accumulation of ERCs, NC-ERCs, and CD73-KO-ERCs in the liver, these cells were labeled with CellTracker™ CM-Dil (Invitrogen, USA) and injected into Con A-induced hepatitis mice (these mice were independent to the main cell therapy experiments). To be specific, the cells were harvested and labeled with CM-Dil solution (2 µM) at 37 ℃ for 5 min and then at 4 ℃ for 15 min. Next, the cells were washed three times and resuspended at a density of 1 × 10^6^ cells/200 µL. Then, these cells were injected through the tail vein into Con A-induced hepatitis mice, respectively. Finally, 24 h later, the livers of those model mice were harvested and imaged by a live animal imaging system.

### Histology, immunohistochemistry, and TUNEL assay

For histology, livers were fixed in 10% formalin, embedded in paraffin, and sliced into 5-µm sections. Hematoxylin and eosin (H&E) staining was then conducted on sections. Confluent liver necrosis was measured on H&E-stained liver sections via ImageJ software (edition 1.53c, National Institutes of Health, USA). Immunohistochemistry was performed using antibodies for CD4 (EPR19514, Abcam, USA). The TUNEL assay was conducted using the One-step TUNEL FITC Apoptosis Detection Kit (APPExBIO, USA) according to the instructions.

### Detection of serum transaminase concentrations

The levels of serum alanine aminotransferase (ALT) and aspartate aminotransferase (AST) were measured utilizing ALT and AST assay kits (Nanjing Jiancheng, Nanjing, China) to evaluate liver function and liver injury.

### Flow cytometry analysis

The flow cytometry analysis was used to identify ERCs and detect the population of T cell subsets in this study. The staining process was the same as we previously mentioned [20]. Briefly, after being separated into single-cell suspension, the collected cells (100 µL/test) were stained with Zombie NIR™ (BioLegend, USA) to identify dead/live cells. And then stained these cells with fluorescent-labeled antibodies, which were purchased from BioLegend and eBioscience, including anti-human: CD45-FITC (Clone: HI30), HLA-DR-FITC (Clone: L243), CD73-FITC (Clone: AD2), CD105-PE-Cyanine 7 (Clone: SN6), and anti-mouse: CD45-PE-Cyanine7 (Clone: 30-F11), CD45-Percp-Cyanine 5.5 (Clone: 30-F11), CD3-FITC (Clone: 145-2C11), CD4-PE (Clone: GK1.5), CD4-FITC (Clone: RM4-5), CD8a-Percp-Cyanine 5.5 (Clone: 53-6.7), CD69-APC (Clone: H1.2F3), CD154-PE (Clone: SA047C3), IFN-γ-PE (Clone: XMG1.2), IL-17A-Percp-Cyanine 5.5 (Clone: eBio17B7), CD25-PE (Clone: PC61.5), Foxp3-APC (Clone: FJK-16s).

### Enzyme-linked immunosorbent assay (ELISA)

The mouse IFN-γ and TNF-α ELISA kits purchased from DAKEWE (Beijing, China) were used to detect the levels of IFN-γ and TNF-α in this study. All experimental procedures were carried out following the manufacturer's handbooks.

### Statistical analysis

All statistics were calculated with GraphPad Prism 9.3.0 (GraphPad Software, LLC), and the obtained experimental data were presented as mean ± SD. For the analysis of differences between multiple groups, a one-way analysis of variance (ANOVA) was used. *P < 0.05; **P < 0.01; ***P < 0.001.

## Results

### Construction and characterization of CD73-KO-ERCs

**Fig. 1:**
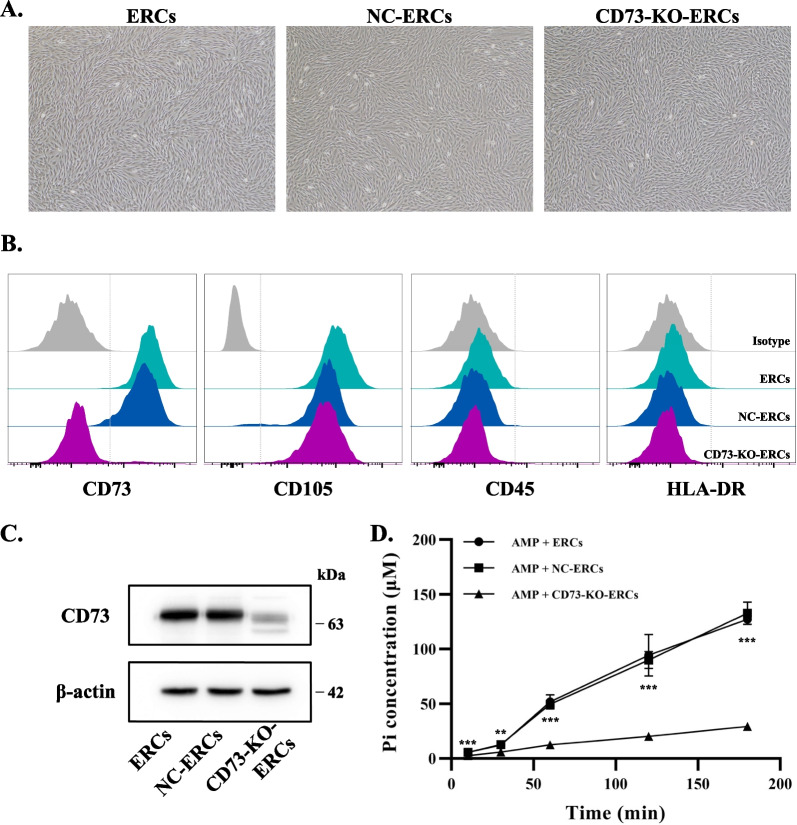
Characterization of ERCs and CD73-KO-ERCs. **A** Morphology of ERCs, and CD73-KO-ERCs at passage 3 (magnification 40 ×). **B** Flow cytometry analysis of stem cell surface markers. **C** Expression of CD73 in ERCs, NC-ERCs and CD73-KO-ERCs by western blot. Full-length blots/gels are presented in Additional file 2: Figure S2. **D** AMPase activity of ERCs, NC-ERCs, and CD73-KO-ERCs. Statistical analysis was done by one-way ANOVA, ***P < 0.001

Knocking out CD73 on the ERCs did not change the morphology, NC-ERCs and CD73-KO-ERCs all showed a fibroblast-like phenotype as ERCs (Fig. 1A). Successful knockout of CD73 (CD73-KO-ERCs) was confirmed by flow cytometry and western blot (Fig. 1B, C). Stem cell surface marker analysis indicated no obvious differences between NC-ERCs and CD73-KO-ERCs other than CD73 (Fig. 1B).

To further determine the successful knockout of CD73 on ERCs, the AMPase activity of CD73 was detected indirectly utilizing the PiColorLock Phosphate Detection Reagent (Abcam, USA). As shown in Fig. 1D, the concentration of free Pi in ERC- or NC-ERC-group showed a sharp rise over time, while the rate of AMP hydrolysis in the CD73-KO-ERC-group was significantly slower (ERC- or NC-ERC-group vs. CD73-KO-ERC-group, *P* < 0.001). This result further clarified the successful knockout of CD73 in CD73-KO-ERCs and the enzymatic function of CD73.

### CD73-mediated AMPase activity is essential for ERCs to inhibit CD4^+^ T cell activation and function in vitro

**Fig. 2:**
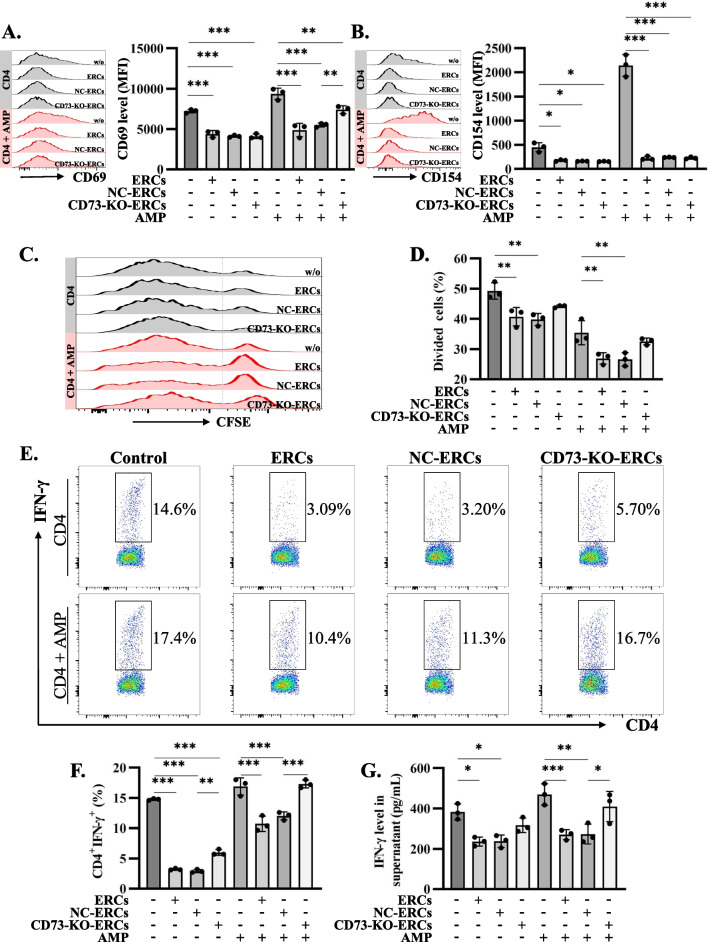
CD73 as AMPase is essential for ERCs in the control of CD4^+^ T cell activation and function. Conventional CD4^+^ T cells were stimulated with αCD3/αCD28 and incubated with ERCs, NC-ERCs, CD73-KO-ERCs, and AMP (50 µM) as indicated. The CD4^+^ T cell activation and function were evaluated after 3 days by flow cytometry and ELISA. **A**, **B** The activation markers CD69 and CD154 were depicted and analyzed (gated on the CD4^+^ population). **C**, **D** The proliferation of CD4^+^ T cells was measured by the dilution of CFSE. **E**, **F** The representative pseudocolor plots and statistical graphs of the CD4^+^IFN-γ^+^ population. **G** IFN-γ production was detected by ELISA in the cell culture supernatant. *n* = 3 per group. Statistical analysis was performed using one-way ANOVA, *P < 0.05; **P < 0.01; ***P < 0.001. Data in bar graphs represent mean ± SD

As shown in Fig. 1B, CD73 is highly expressed on ERCs. We hypothesized that the CD73-mediated AMPase activity derived from ERCs contributes to ADO production and immune suppression. To test this, we stimulated CD4^+^ conventional T cells and added ERCs with different pretreatments. As shown in Fig. 2A and B, co-culture with three kinds of ERCs all significantly reduce the expression of T cell activation markers CD69 and CD154 (CD69: *P* < 0.001; CD154: *P* < 0.05). It is worth noticing that when exogenous AMP was introduced to the culture medium to ensure sufficient substrate for CD73 in all situations, the reduction of CD69 expression on CD4^+^ T cells was more apparent when cultured with NC-ERCs than CD73-KO-ERCs (NC-ERCs vs*.* CD73-KO-ERCs, *P* < 0.01. Figure 2A). In addition, we also detected the proliferation of CD4^+^ T cells in different groups. As we expected, ERCs or NC-ERCs obviously inhibited the proliferation of CD4^+^ T cells either in the presence of AMP or not while CD73-KO-ERCs did not (ERCs vs*.* w/o, *P* < 0.01; NC-ERCs vs*.* w/o, *P* < 0.01. Figure 2C, D). Furthermore, we measured the population of CD4^+^IFN-γ^+^ Th1 cells by flow cytometry and IFN-γ levels in the supernatants by ELISA. As shown in Fig. 2E–G, ERCs or NC-ERCs significantly inhibited the differentiation of activated CD4^+^ T cells to Th1 cells compared with CD73-KO-ERCs, as well as the production of IFN-γ, especially in the presence of AMP. Those results imply that the expression of CD73 on ERCs can mediate the inhibition of activation, proliferation, differentiation, and cytokine production of conventional CD4^+^ T cells, most likely through the degradation of AMP to immunosuppressive ADO.

### Knocking out CD73 impaired the therapeutic efficacy of ERCs against Con A-induced hepatitis

**Fig. 3:**
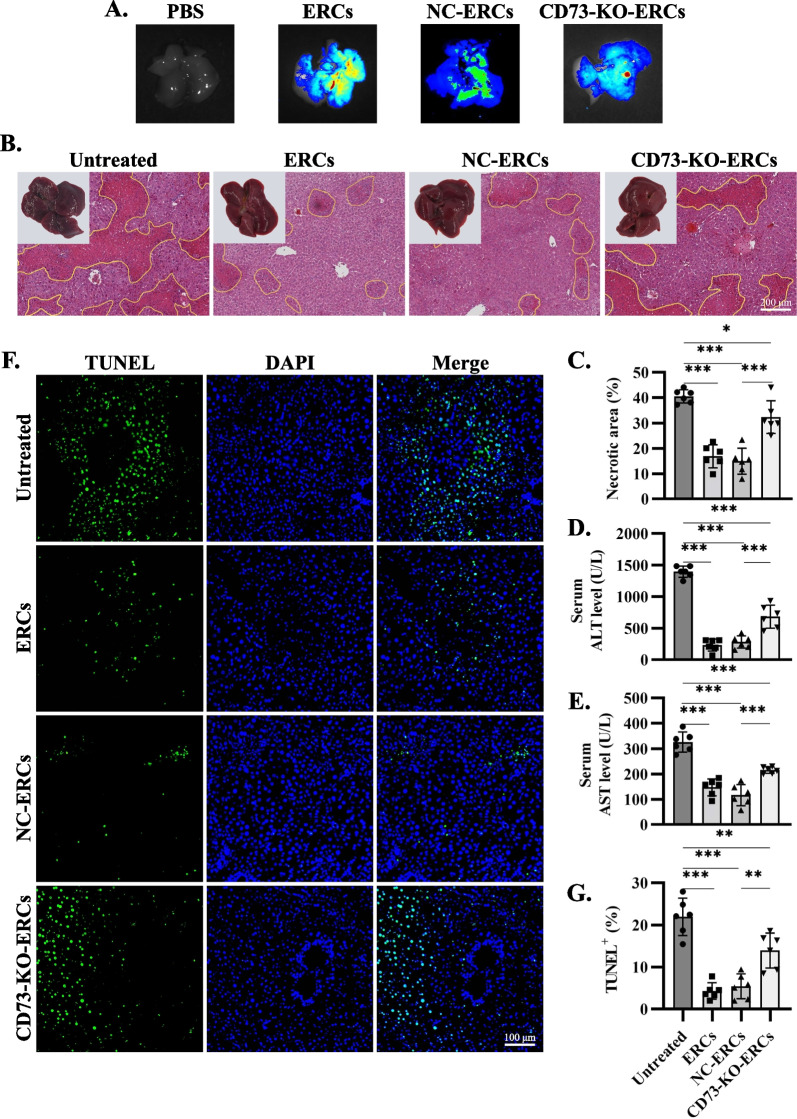
CD73 mediated the therapeutic efficacy of ERCs against Con A-induced hepatitis. To assess the cell-homing ability between ERCs and CD73-KO-ERCs, these cells were labeled with CM-Dil and injected intravenously into the Con A-induced hepatitis mice. **A** 24 h later, the livers of these mice were isolated and observed under a live animal imaging system. To assess the influence of CD73 deletion on ERCs’ therapeutic efficiency in Con A-induced hepatitis, **B** macroscopic appearance and confluent liver necrosis (yellow lines) of representative livers after ERCs, NC-ERCs, and CD73-KO-ERCs treatment were identified on H&E staining. **C** Necrotic areas were quantified as [%] of liver section area. Concentrations of serum **D** alanine transaminase (ALT) and **E** aspartate aminotransferase (AST) in different groups are depicted. **F** Representative TUNEL staining of livers section from Con A-treated mice with the indicated treatments (green), nuclei were counterstained with DAPI (blue). **G** The statistical graphs of TUNEL staining. *n* = 6 per group. One-way ANOVA was used for statistical analysis, **P < 0.01; ***P < 0.001. Data in bar graphs represent mean ± SD

As shown in Fig. 3A, injected ERCs and CD73-KO-ERCs could migrate to the injured liver. To analyze the importance of CD73 for the immunomodulatory effects of ERCs in acute hepatitis, Con A was injected and different interventions were administrated. Liver necrosis was macroscopically apparent in the untreated group 24 h after Con A injection, in contrast, ERC-treated livers were indistinguishable from NC-ERC- and CD73-KO-ERC-treated groups (Fig. 3B). Histological analysis revealed that liver necrosis was significantly reduced in ERC-treated group compared with untreated group, and no difference between ERC-treated or NC-ERC-treated groups (ERC-treated group vs. untreated group, *P* < 0.001; NC-ERC-treated group vs*.* untreated group, *P* < 0.001. Figure 3B, C). It was worth noting that more necrosis was found in CD73-KO-ERC-treated group when compared with NC-ERC-treated groups (CD73-KO-ERC-treated group vs*.* NC-ERC-treated group, *P* < 0.001. Figure 3B, C). In addition, alleviated liver damage in ERC-treated group was also confirmed by prominently reduced serum transaminase concentrations, while there was a marked elevation in CD73-KO-ERC-treated group compared with NC-ERC-treated group (Fig. 3D, E). To further confirm these differences in cell death, TUNEL assay revealed confluent areas of apoptotic hepatocytes in untreated livers, while only scattered apoptotic cells were apparent in ERC-treated or NC-ERC-treated livers (ERC-treated group vs. untreated group, *P* < 0.001; NC-ERC-treated group vs. untreated group, *P* < 0.001. Figure 3F, G). As same as the H&E results, more apoptotic hepatocytes were found in CD73-KO-ERC-treated livers than those in NC-ERC-treated livers (CD73-KO-ERC-treated group vs*.* NC-ERC-treated group, *P* < 0.01. Figure 3F, G). These data imply that ERCs exhibited significant therapeutic effects in Con A-induced hepatitis and the knockout of CD73 remarkably impaired the therapeutic efficacy of ERCs.

### CD73 expressing ERCs modulated the liver immune microenvironment

**Fig. 4:**
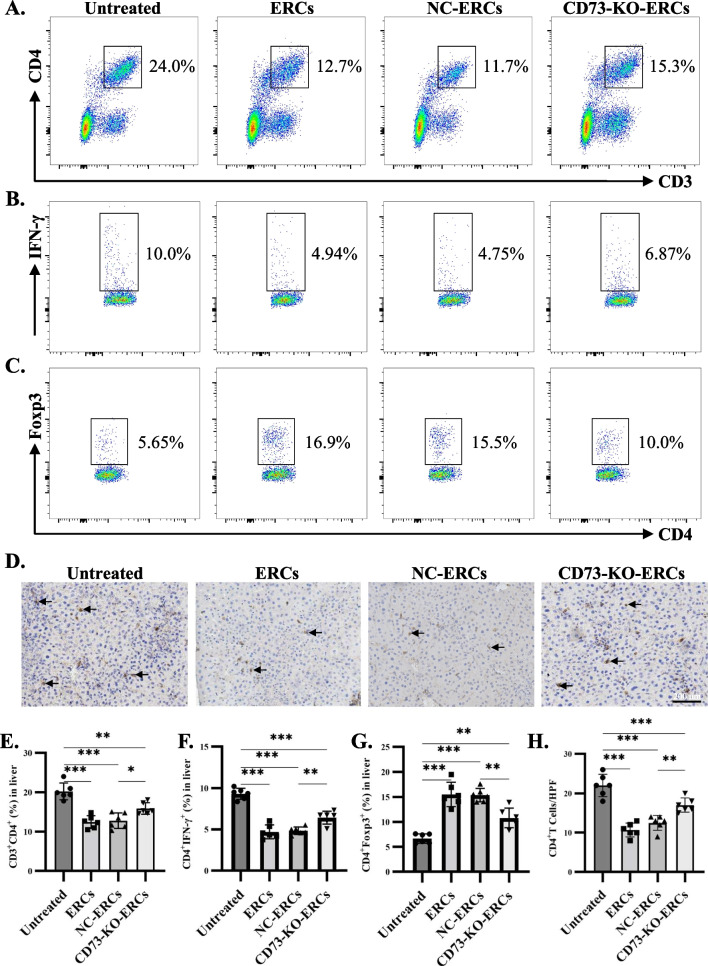
CD73 expressing ERCs modulated the hepatic local immune microenvironment. 24 h after model induction, liver tissue single-cell suspensions were made and mononuclear cells were isolated and stained with fluorochrome-conjugated antibodies, and then detected the CD4^+^ T cell subsets by flow cytometry (*n* =6 per group). The representative pseudocolor plots of **A** CD3^+^CD4^+^ cells, **B** CD4^+^IFN-γ^+^ cells, and **C** CD4^+^Foxp3^+^ cells were depicted. We also detected the CD4^+^ cells by immunohistochemistry (*n* = 6 per group), and **D** the representative images were exhibited. The positive staining area was shown by an arrow. **E–H** The percentage of CD3^+^CD4^+^, CD4^+^IFN-γ^+^ Th1 and CD4^+^Foxp3^+^ Tregs detected by flow cytometry or immunohistochemistry from liver tissues of different groups were analyzed. Data were dealt with one-way ANOVA. *P < 0.05; **P < 0.01; ***P < 0.001. Data in bar graphs represent mean ± SD

Excessive ATP in the extracellular component can promote the proliferation of CD4^+^ conventional T cells, while ADO inhibits the activation of conventional CD4^+^ T cells [11], which arises the question that whether CD73, the critical enzyme of AMP to ADO, participates in the ERC-mediated immunoregulatory effects in the liver. To further explore this issue, mononuclear cells were isolated from livers and detected by flow cytometry. CD3^+^CD4^+^ and CD3^+^CD8^+^ T cell subsets were identified. The proportion of infiltrating CD3^+^CD4^+^ T cells was profoundly decreased in ERC- or NC-ERC-treated mouse livers (ERC-treated group vs*.* untreated group, *P* < 0.001; NC-ERC-treated group vs*.* untreated group, *P* < 0.001. Figure 4A, E). Though the CD73-KO-ERC-treated group also achieved a low infiltration of CD3^+^CD4^+^ T cells in the liver, they had weakened effects than the NC-ERC-treated group (CD73-KO-ERC-treated group vs*.* untreated group, *P* < 0.01; CD73-KO-ERC-treated group vs*.* NC-ERC-treated group, *P* < 0.05). There is no significant difference in the CD3^+^CD8^+^ T cell subsets between the four groups (shown in Additional file 3: Figure S3). This finding was also confirmed by immunohistochemistry (Fig. 4D, H). Helper CD4^+^ T cells play a critical role in the pathogenesis of Con A-induced hepatitis, so we further identified the proportion of liver-infiltrating Th1 (CD4^+^IFN-γ^+^) and Th17 (CD4^+^IL-17A^+^) subsets. As shown in Fig. 4B, F, ERC- and NC-ERC-treated groups achieved less infiltration of Th1 cells than the untreated group, though CD73-KO-ERC-treated group also has less proportion of Th1 cells than the untreated group, they were higher than the NC-ERC-treated group. There is no significant difference in the Th17 cell subsets between these groups (shown in Additional file 4: Figure S4). Furthermore, we have also detected the regulatory CD4^+^ T cells, as shown in Fig. 4C, G, ERC-treated or NC-ERC-treated group achieved a high proportion of CD4^+^Foxp3^+^ Tregs, while the knockout of CD73 dampens the induction of Tregs. Taken together, the above data suggest that CD73 expression on ERCs plays a pivotal role in the regulation of local CD4^+^ T cell response in Con A-induced hepatitis.

### CD73 expressing ERCs inhibited the proliferation of splenic CD4^+^ T cells

**Fig. 5:**
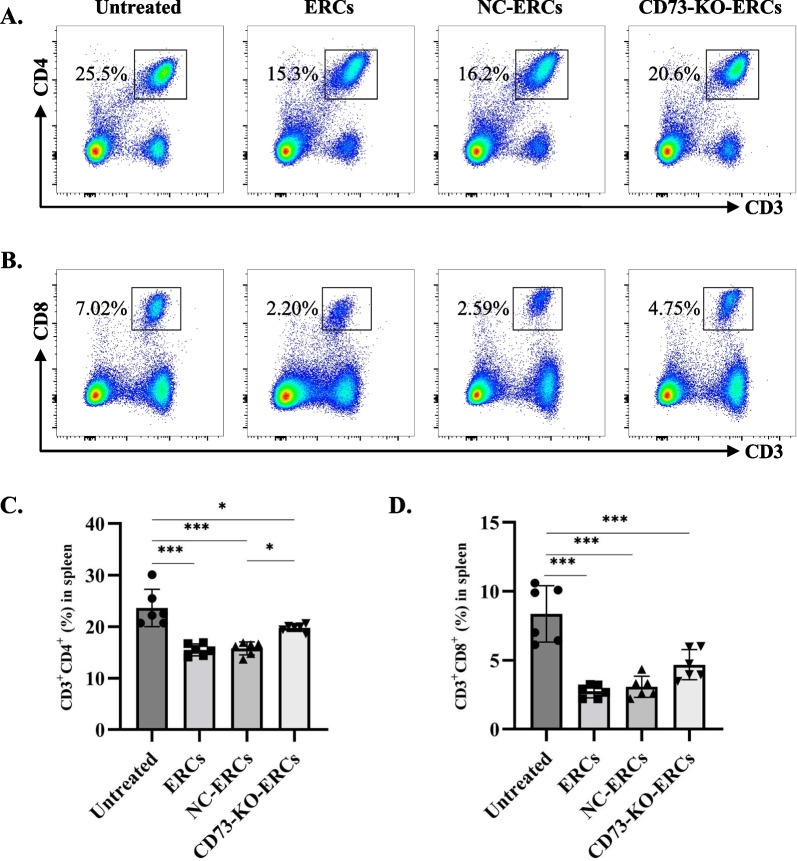
CD73 expression on ERCs reduced the splenic CD4^+^ T cell populations in the treatment of Con A-induced hepatitis. To evaluate the systemic T cell subsets population in different groups, we made the single-cell suspension of splenocytes and detected them by flow cytometry (*n* = 6 per group). **A** Representative pseudocolor plots depict the percentage of CD3^+^CD4^+^ T cells. **B** Representative pseudocolor plots depict the percentage of CD3^+^CD8^+^ T cells. **C** and **D** show the statistical graph. One-way ANOVA was used for statistical analysis, *P < 0.05; ***P < 0.001. Data in bar graphs represent mean ± SD

In addition to the detection of local immune regulatory effects of ERCs, we next analyzed the proportions of CD3^+^CD4^+^ or CD3^+^CD8^+^ T cell subsets in the spleen by flow cytometry. As shown in Fig. 5A–D, the proportions of CD3^+^CD4^+^or CD3^+^CD8^+^ T cells in the ERC-or NC-ERC-treated group were significantly lower than those of untreated group (CD3^+^CD4^+^ T cells: *P* < 0.001; CD3^+^CD8^+^ T cells: *P* < 0.001). Consistent with the previous results, the CD3^+^CD4^+^ T cell populations were higher in the CD73-KO-ERC-treated group when compared with NC-ERC-treated group, though CD73-KO-ERCs also perform an inhibitory effect when compared with the untreated group (CD73-KO-ERC-treated group vs*.* NC-ERC-treated group, *P* < 0.05; CD73-KO-ERC-treated group vs*.*untreated group, *P* < 0.05. Figure 5A, C). There was no statistical difference between CD73-KO-ERC-treated group and NC-ERC-treated group in the population of CD3^+^CD8^+^ T cells (Fig. 5B, D). Collectively, these data suggest that CD73 plays a significant role in the immunoregulatory capacity of ERCs in downregulating the population of splenic CD4^+^ T cells in Con A-induced hepatitis.

### CD73 expressing ERCs inhibited Th1 and Th17 cell differentiation and increased the generation of Tregs in Con A-induced hepatitis mice

**Fig. 6:**
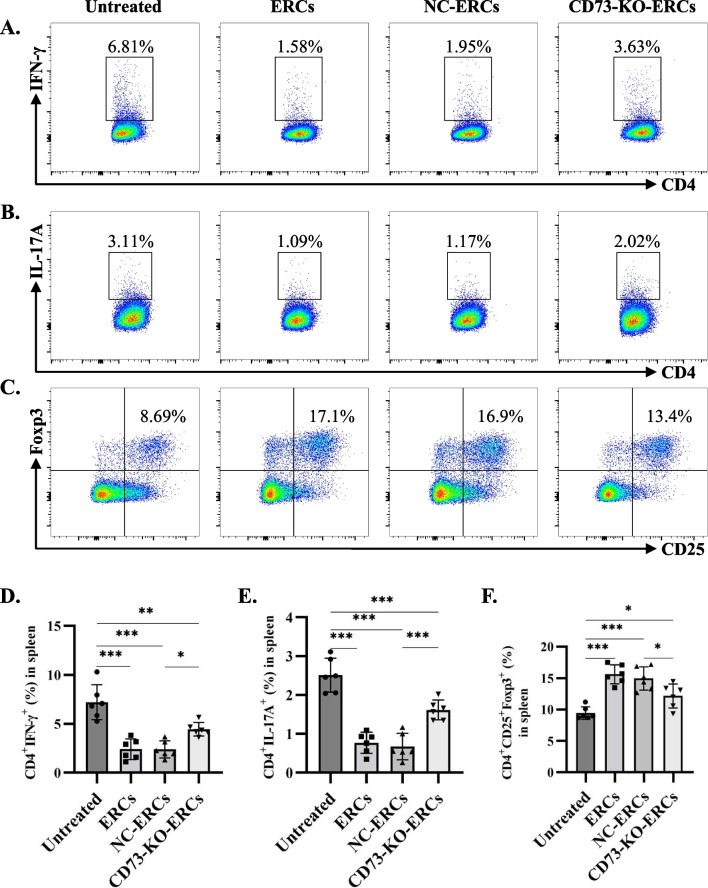
CD73 expression on ERCs reduced splenic Th1 and Th17 populations while increasing the generation of Tregs in the treatment of Con A-induced hepatitis. Splenocytes obtained from the model mice of each group were stained with anti-mouse CD4+IFN-γ, CD4+IL-17A, and CD4+CD25+Foxp3 antibodies, respectively (*n* = 6 per group). The representative pseudocolor plots of **A** Th1 cells (CD4^+^IFN-γ^+^), **B** Th17 cells (CD4^+^IL-17A^+^), and **C** Tregs (CD4^+^CD25^+^Foxp3^+^) were exhibited. The percentage of **D** Th1 cells, **E** Th17 cells, and **F** Tregs were analyzed by one-way ANOVA.*P < 0.05; **P < 0.01; ***P < 0.001. Data in bar graphs represent mean ± SD

CD4^+^ T cell is important for Con A-induced hepatitis [19], the proportions of pro-inflammatory CD4^+^ T cell subsets in splenocytes were further analyzed by flow cytometry, including Th1 (CD4^+^IFN-γ^+^) and Th17 (CD4^+^IL-17A^+^) cells. As shown in Fig. 6A, D, the percentage of Th1 cells was significantly lower in the ERC-treated group compared with that of the untreated group (*P* < 0.001). No obvious difference in the Th1 cell population was found between the ERC-treated group and the NC-ERC-treated group, but a significant elevation was found in the CD73-KO-ERC-treated group compared with those of the NC-ERC-treated group (CD73-KO-ERC-treated group vs. NC-ERC-treated group, *P* < 0.05). Consistently, the same trend was also observed in Th17 subsets (NC-ERC-treated group vs*.* untreated group, *P* < 0.001; CD73-KO-ERC-treated group vs*.* NC-ERC-treated group, *P* < 0.001. Figure 6B, E).

Tregs were generally believed to play a significant role in the maintenance of liver self-tolerance [1]. In the present study, the proportion of Tregs in the spleen was detected. As shown in Fig. 6C and F, the proportion of Tregs was the highest in the ERC-treated group, while an obvious reduction was observed in the CD73-KO-ERC-treated group, though they both induced more Tregs than the untreated group (ERC-treated group vs*.* untreated group, *P* < 0.001; NC-ERC-treated group vs*.* untreated group, *P* < 0.001; CD73-KO-ERC-treated group vs*.* untreated group, *P* < 0.05; CD73-KO-ERC-treated group vs*.* NC-ERC-treated group, *P* < 0.05), indicating that CD73 expressing on ERCs plays a critical role in the promotion of Treg generation.

Collectively, these results reveal that CD73 expression is essential for the immune regulation ability of ERCs. In Con A-induced hepatitis mice, CD73 expressing ERCs reduced CD4^+^ T cell infiltration in livers, and inhibited Th1 and Th17 cell differentiation in splenocytes while promoting the generation of Tregs.

### CD73 expressing ERCs further regulated the levels of pro-inflammatory cytokines in the livers and sera

**Fig. 7:**
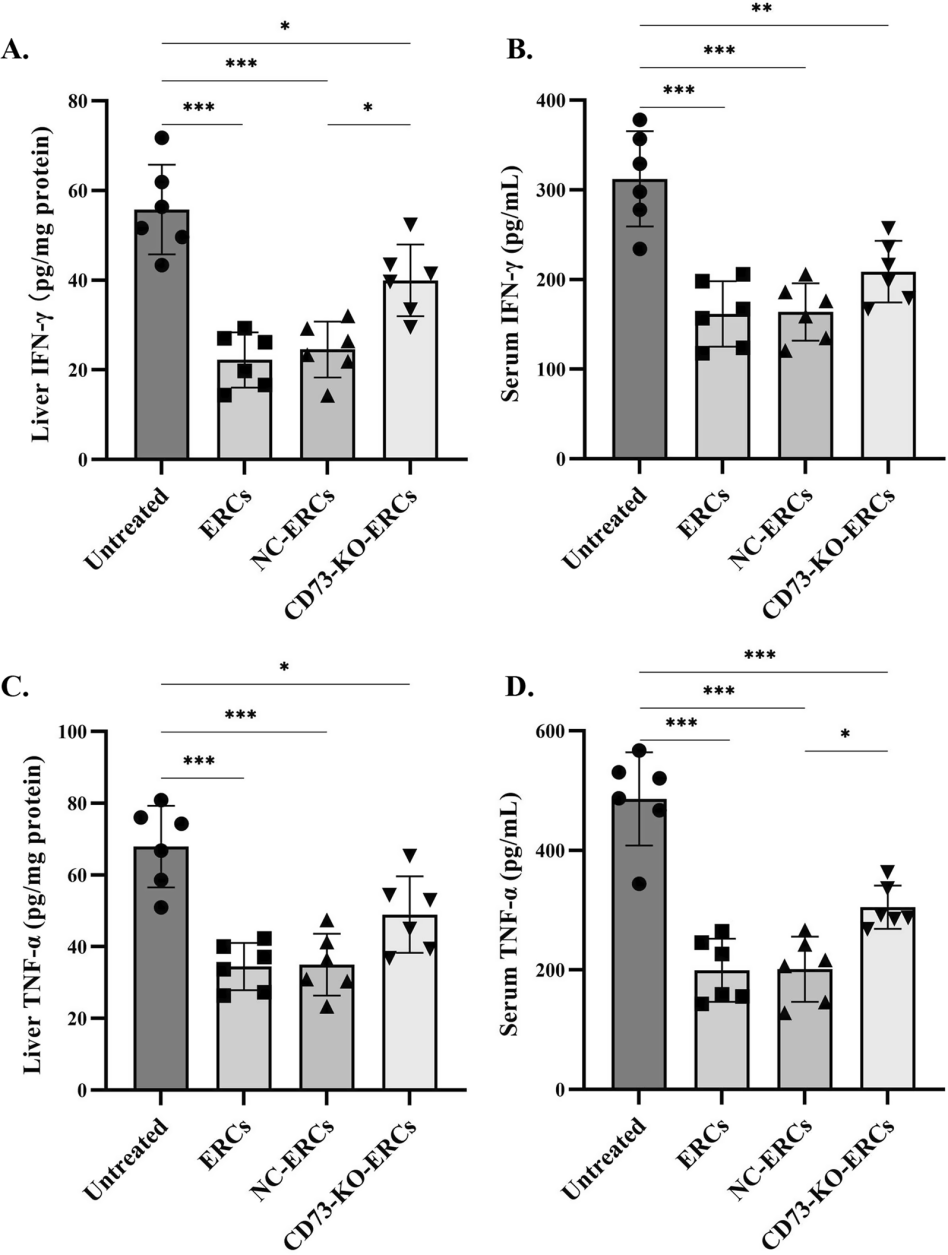
CD73 expression on ERCs reduced the serum levels of pro-inflammatory cytokines in the treatment of Con A-induced hepatitis. To evaluate the CD73 expression of ERCs on the overall function of the model mice’s immune system, the production of pro-inflammatory cytokines (IFN-γ and TNF-α) in the liver tissues and sera was detected via ELISA assay. The liver tissue and serum samples were collected from mice of each group after 24 h of Con A injection (*n* = 6 per group). Here, the levels of IFN-γ in the liver tissue (**A**) and serum (**B**), as well as the levels of TNF-α in the liver tissue (**C**) and serum (**D**), are shown, respectively. Data were analyzed using one-way ANOVA, *P < 0.05; **P < 0.01; ***P < 0.001. Data in bar graphs represent mean ± SD

To determine whether CD73 expression of ERCs plays a role in regulating cytokines in Con A-induced hepatitis, we detected the levels of IFN-γ and TNF-α in the livers and sera from different groups. As shown in Fig. 7, the levels of pro-inflammatory cytokines (IFN-γ, TNF-α) were significantly reduced in the livers and sera after treatment with ERCs (liver: IFN-γ: ERC-treated group vs*.* untreated group, *P* < 0.001; TNF-α: ERC-treated group vs*.* untreated group, *P* < 0.001; serum: IFN-γ: ERC-treated group vs*.* untreated group, *P* < 0.001; TNF-α: ERC-treated group vs*.* untreated group, *P* < 0.001). The ERCs transfected with empty lentivirus did not alter the cytokine profiles both in the liver and serum compared with the ERC-treated group (IFN-γ and TNF-α: NC-ERC-treated group vs*.* ERC-treated group, no significance). However, the IFN-γ and TNF-α levels showed an obvious elevation in the CD73-KO-ERCs-treated group when compared with the NC-ERC-treated group (liver: IFN-γ: CD73-KO-ERC-treated group vs*.* NC-ERC-treated group, *P* < 0.05; serum: TNF-α: ERC-treated group vs*.* untreated group, *P* < 0.05). These results show that ERCs possess the ability to downregulate the profile of pro-inflammatory cytokines and the CD73 expression is critical for ERCs to maintain this property.

## Discussion

Con A-induced acute hepatitis is widely recognized as T cell-mediated hepatitis [2, 21, 22]. Acute liver damage and systemic immunological activation brought by intravenous injection of Con A in mice are similar to the pathophysiology of immune-mediated hepatitis in humans [19]. In the present study, we first demonstrated that CD73 mediates the therapeutic efficiency of ERCs in Con A-induced hepatitis. We showed that the deletion of CD73 on ERCs weakened its ability to inhibit the CD4^+^ T cell activation and function in vitro and led to poor therapeutic efficiency of ERCs in Con A-induced hepatitis which was associated with extensive liver necrosis, massive infiltration of effector CD4^+^ T cells, decreased generation of Tregs, and elevated tissue and serum levels of pro-inflammatory cytokines (IFN-γ, TNF-α).

CD73 is a key enzyme in purinergic metabolism with the function of dephosphorylating immune-stimulating AMP to immune-suppressive ADO and has been demonstrated to play a key regulatory role in several pathological and physiological processes [23, 24]. As a stem cell marker, CD73 is widely distributed on ERCs [12]. Herein, we knocked out the *NT5E* gene which coded CD73 in ERCs by CRISPR/Cas-9 system, and identified using flow cytometry and western blot. In addition, the results of the CD73 enzyme function test further proved the successful knockout of CD73 on the one hand and demonstrated the high efficiency of CD73 in catalyzing AMP to ADO on the other hand.

Activated T lymphocytes play a crucial role in Con A-induced liver injury [25–27]. CD4^+^ T cells infiltrate into the liver tissue and produce large amounts of inflammatory cytokines, such as IFN-γ, TNF-α, IL-2, and IL-6 [28–30]. In the present study, we found that ERCs could significantly inhibit the enhancing immunity function of CD4^+^ T cells both in vitro and in Con A-induced hepatitis response, while there was an apparent recovery when CD73 was knocked out on ERCs. During the occurrence of liver injury, the impaired cells release numerous amounts of ATP into the local milieus and induce the activation and immune response of CD4^+^ T cells [6, 11]. In the physiological state, the enhanced ATP production is followed by its sequential dephosphorylation to AMP by CD39, and to ADO by CD73 [24, 31]. As an immune-suppressive molecule, ADO activates the A_2A_ receptors (A_2A_R) expressed on T effector cells and then inhibits the excessive immune response [11, 32]. However, in inflammation conditions, when naive T cells activate, the CD73 expression on CD4^+^ T cells is downregulated to prevent autocrine/paracrine immune-suppressive adenosine generation [33]. Considering the accumulation of ERCs in impaired livers in this study, we speculated that the highly expressed CD73 on ERCs participates in the degradation of immune-stimulatory ATP, which may maintain the balance of purinergic metabolism in inflammation sites and explain the different fates of CD4^+^ conventional T cells in the ERC-treated and CD73-KO-ERC-treated groups.

Tregs are pivotal for the maintenance of immunological self-tolerance and the prevention of autoimmunity [34, 35]. In humans, the reduced number and functional defects of Tregs favor the development of an excessive immune response to self-antigens and cause AIH in genetically predisposed individuals [36, 37]. So, in addition to conventional CD4^+^ T cell subsets, we have also detected the population of hepatic and splenic Tregs in different groups in the present study. Similar to our and other researchers’ previous study, ERCs could effectively enhance the population of Tregs in inflammatory conditions [38–40]. Interestingly, a remarkable reduction of the Treg population was found in the CD73-KO-ERC-treated group when compared with the ERC-treated group. Ohta et al*.* [41] highlighted a self-reinforcing loop driven by ADO generation in the immunosuppressive activity of Tregs. To be specific, the activation of A_2A_R expressed on Tregs facilitated the expansion of these cells, and in turn, an increased immunoregulatory ability was acquired [41]. The significance of this signaling loop has been confirmed in the protection of ischemia and reperfusion-induced inflammation and injury to the kidney [42]. Based on our results, we speculate that the CD73 derived from ERCs also effectively generates ADO in inflammation sites and promotes the expansion of immunoregulatory Tregs, which strongly mediates the immunomodulatory effects of ERCs.

The intrahepatic IFN-γ and TNF-α are crucial proinflammatory cytokines for inducing hepatocyte apoptosis and hepatic fibrosis [43]. Intriguingly, our data in this study showed that the profile of IFN-γ and TNF-α was dramatically raised in the liver tissue and serum of untreated mice, and a significant reduction was observed in the ERC-treated group, while the deletion of CD73 in ERCs impaired their immunosuppressive effects. IFN-γ and TNF-α are mainly produced by Th1 cells and M1 macrophages [44, 45]. So, the present tendency in cytokines was consistent with our flow cytometry results about Th1 cells.

In the present study, some underlying mechanisms need to be discussed. On the one hand, CD73 expression improves the immunosuppressive ability of ERCs which is characterized by anti-infiltration and proliferation of pro-inflammatory CD4^+^ T conventional cells and reduced production of pro-inflammatory cytokines. On the other hand, CD73 expression of ERCs promotes the generation of immunoregulatory Tregs. However, there are also some limitations in the present study. First, due to ERCs are not suitable for monoclonal culturing, it is impossible to fully knock out CD73 in the ERCs, so there is still some CD73 expression in the CD73-KO-ERCs as shown in our flow cytometry and western blot results. These remained CD73 may partially explain the incompletely recovery in the CD73-KO-ERC-treated group when compared with NC-ERC-treated group. To solve this problem, we will consider extracting ERCs from CD73 deficient mice for further study [46, 47]. In addition, the powerful immunomodulatory properties of ERCs are the comprehensive result of various immunomodulatory molecules produced by it, such as PD-L1 [48], PGE-2 [17], Gal 9 [15, 49], and so on, we cannot exclude the influence of other immunomodulatory molecules. Finally, the direct evidence and specific pathway of CD73 expression on ERCs regulates the local purinergic signaling (cell-to-cell direct contact or through paracrine function such as exosomes) need to be further explored.

## Conclusion

In conclusion, CD73 is critical for ERC-mediated therapeutic efficiency in the treatment of Con A-induced hepatitis. The findings of this study serve as the foundation for the clinical use of ERCs not only in autoimmune hepatitis, but also in other immune-mediated diseases.

### Supplementary Information

**Additional file 1: Figure S1.** The purity of the isolated CD4^+^ cells was assessed by flow cytometry, and the percentage of live CD4^+^ cells achieved 97.1%.

**Additional file 2: Figure S2.** The Full-length merge blots of CD73 and β-actin in immunoblotting (protein). The shiny images used in the manuscript corresponded to the part marked here by the black frame. The lanes on the right above were duplicates. **(A)** The merge blots of C73. **(B)** The merge blots of β-actin.

**Additional file 3: Figure S3.** The liver infiltration of CD8^+^ T cells in different groups. **(A)** The representative pseudocolor plots were depicted. **(B)** show the statistical graph. One-way ANOVA was used for statistical analysis, no significance was found between those groups. Data in bar graphs represent mean ± SD.

**Additional file 4: Figure S4.** The liver infiltration of CD4^+^IL-17A^+^ Th17 cells in different groups. **(A)** The representative pseudocolor plots were depicted. **(B)** show the statistical graph. One-way ANOVA was used for statistical analysis, no significance was found between those groups. Data in bar graphs represent mean ± SD.

## Data Availability

All data generated or analyzed during this study are included in this published article or are available from the corresponding author on reasonable request.

## Abbreviations

ATP: Adenosine triphosphate
AMP: Adenosine monophosphate
ADO: Adenosine
Con A: Concanavalin A
CD73: Ecto-5`-nucleotidase
CD73-KO-ERCs: ERCs knockout of *NT5E* gene by lentivirus transfection
ERCs: Endometrial regenerative cells
IDO: Indoleamine 2,3 dioxygenase
MSCs: Mesenchymal stem/stromal cells
NC-ERCs: ERCs transfected with blank lentivirus
P1R: Purinergic type 1 receptors
P2R: Purinergic type 2 receptors
PG-E2: Prostaglandin E2
PD-L1: Programmed cell death 1 ligand 1
TUNEL: Terminal deoxynucleotidyl transferase-mediated dUTP nick-end labeling

## References

[1] Mieli-Vergani G, Vergani D, Czaja AJ, Manns MP, Krawitt EL, Vierling JM, Lohse AW, Montano-Loza AJ (2018). Autoimmune hepatitis. Nat Rev Dis Primers.

[2] Volarevic V (2012). Protective role of IL-33/ST2 axis in Con A-induced hepatitis. J Hepatol.

[3] Shuai Z (2016). Adaptive immunity in the liver. Cell Mol Immunol.

[4] Robinson MW, Harmon C, O'Farrelly C (2016). Liver immunology and its role in inflammation and homeostasis. Cell Mol Immunol.

[5] Cekic C, Linden J (2016). Purinergic regulation of the immune system. Nat Rev Immunol.

[6] Junger WG (2011). Immune cell regulation by autocrine purinergic signalling. Nat Rev Immunol.

[7] Eltzschig HK, Sitkovsky MV, Robson SC (2012). Purinergic signaling during inflammation. N Engl J Med.

[8] Ayata CK (2012). Purinergic P2Y(2) receptors promote neutrophil infiltration and hepatocyte death in mice with acute liver injury. Gastroenterology.

[9] Deaglio S (2007). Adenosine generation catalyzed by CD39 and CD73 expressed on regulatory T cells mediates immune suppression. J Exp Med.

[10] Borg N (2017). CD73 on T cells orchestrates cardiac wound healing after myocardial infarction by purinergic metabolic reprogramming. Circulation.

[11] Antonioli L (2013). CD39 and CD73 in immunity and inflammation. Trends Mol Med.

[12] Meng X (2007). Endometrial regenerative cells: a novel stem cell population. J Transl Med.

[13] Chen L (2019). Menstrual blood-derived stem cells: toward therapeutic mechanisms, novel strategies, and future perspectives in the treatment of diseases. Stem Cell Res Ther.

[14] Li G (2021). IL-37 overexpression enhances therapeutic effect of endometrial regenerative cells in concanavalin A-induced hepatitis. Cytotherapy.

[15] Wang H (2021). Endometrial regenerative cells with galectin-9 high-expression attenuate experimental autoimmune hepatitis. Stem Cell Res Ther.

[16] Zhang F (2022). Comprehensive immune cell analysis of human menstrual-blood-derived stem cells therapy to concanavalin A hepatitis. Front Immunol.

[17] Luz-Crawford P (2016). The immunosuppressive signature of menstrual blood mesenchymal stem cells entails opposite effects on experimental arthritis and graft versus host diseases. Stem Cells.

[18] Gargett CE, Schwab KE, Deane JA (2016). Endometrial stem/progenitor cells: the first 10 years. Hum Reprod Update.

[19] Tiegs G, Hentschel J, Wendel A (1992). A T cell-dependent experimental liver injury in mice inducible by concanavalin A. J Clin Investig.

[20] Qin H (2022). IL-37 overexpression promotes endometrial regenerative cell-mediated inhibition of cardiac allograft rejection. Stem Cell Res Ther.

[21] Hanson JC (2006). Transgenic overexpression of interleukin-8 in mouse liver protects against galactosamine/endotoxin toxicity. J Hepatol.

[22] Wolf AM (2006). Up-regulation of the anti-inflammatory adipokine adiponectin in acute liver failure in mice. J Hepatol.

[23] Vaughn BP, Robson SC, Burnstock G (2012). Pathological roles of purinergic signaling in the liver. J Hepatol.

[24] Vuerich M, Robson SC, Longhi MS (2019). Ectonucleotidases in intestinal and hepatic inflammation. Front Immunol.

[25] Béland K (2015). Depletion of B cells induces remission of autoimmune hepatitis in mice through reduced antigen presentation and help to T cells. Hepatology.

[26] Zheng C (2018). CD24 aggravates acute liver injury in autoimmune hepatitis by promoting IFN-γ production by CD4(+) T cells. Cell Mol Immunol.

[27] Lohse AW, Dienes HP, Meyer zum Büschenfelde KH (1998). Suppression of murine experimental autoimmune hepatitis by T-cell vaccination or immunosuppression. Hepatology.

[28] Gantner F (1995). Concanavalin A-induced T-cell-mediated hepatic injury in mice: the role of tumor necrosis factor. Hepatology.

[29] Küsters S (1996). Interferon gamma plays a critical role in T cell-dependent liver injury in mice initiated by concanavalin A. Gastroenterology.

[30] Mizuhara H (1996). Critical involvement of interferon gamma in the pathogenesis of T-cell activation-associated hepatitis and regulatory mechanisms of interleukin-6 for the manifestations of hepatitis. Hepatology.

[31] Savio LEB, Robson SC, Longhi MS (2020). Ectonucleotidase modulation of lymphocyte function in gut and liver. Front Cell Dev Biol.

[32] Chouker A (2008). Critical role of hypoxia and A2A adenosine receptors in liver tissue-protecting physiological anti-inflammatory pathway. Mol Med.

[33] Regateiro FS, Cobbold SP, Waldmann H (2013). CD73 and adenosine generation in the creation of regulatory microenvironments. Clin Exp Immunol.

[34] Baecher-Allan C, Hafler DA (2006). Human regulatory T cells and their role in autoimmune disease. Immunol Rev.

[35] Wing JB, Tanaka A, Sakaguchi S (2019). Human FOXP3(+) regulatory T cell heterogeneity and function in autoimmunity and cancer. Immunity.

[36] Manns MP, Lohse AW, Vergani D (2015). Autoimmune hepatitis–update 2015. J Hepatol.

[37] Krawitt EL (2006). Autoimmune hepatitis. N Engl J Med.

[38] Lv Y (2014). Endometrial regenerative cells as a novel cell therapy attenuate experimental colitis in mice. J Transl Med.

[39] Lan X (2017). Stromal cell-derived factor-1 mediates cardiac allograft tolerance induced by human endometrial regenerative cell-based therapy. Stem Cells Transl Med.

[40] Aleahmad M (2021). Endometrial mesenchymal stem/stromal cells: the Enigma to code messages for generation of functionally active regulatory T cells. Stem Cell Res Ther.

[41] Ohta A (2012). The development and immunosuppressive functions of CD4(+) CD25(+) FoxP3(+) regulatory T cells are under influence of the adenosine-A2A adenosine receptor pathway. Front Immunol.

[42] Kinsey GR (2012). Autocrine adenosine signaling promotes regulatory T cell-mediated renal protection. J Am Soc Nephrol.

[43] Feng XX (2019). IL-37 suppresses the sustained hepatic IFN-γ/TNF-α production and T cell-dependent liver injury. Int Immunopharmacol.

[44] Kremer M (2006). Favored T helper 1 response in a mouse model of hepatosteatosis is associated with enhanced T cell-mediated hepatitis. Hepatology.

[45] Schümann J (2000). Importance of Kupffer cells for T-cell-dependent liver injury in mice. Am J Pathol.

[46] Stagg J (2011). CD73-deficient mice have increased antitumor immunity and are resistant to experimental metastasis. Cancer Res.

[47] Deane JA (2016). The mouse endometrium contains epithelial, endothelial and leucocyte populations expressing the stem cell marker telomerase reverse transcriptase. Mol Hum Reprod.

[48] Ye K (2018). B7–H1 expression is required for human endometrial regenerative cells in the prevention of transplant vasculopathy in mice. Stem Cells Int.

[49] Zhao Y (2020). Galectin-9 is required for endometrial regenerative cells to induce long-term cardiac allograft survival in mice. Stem Cell Res Ther.

