# Supplementary figures and images for: CD73 mediates the therapeutic effects of endometrial regenerative cells in concanavalin A-induced hepatitis by regulating CD4+ T cells
Source: Stem Cell Res Ther. 2023 Sep 29;14:277. doi: 10.1186/s13287-023-03505-2 (PMC10543328; doi:10.1186/s13287-023-03505-2)

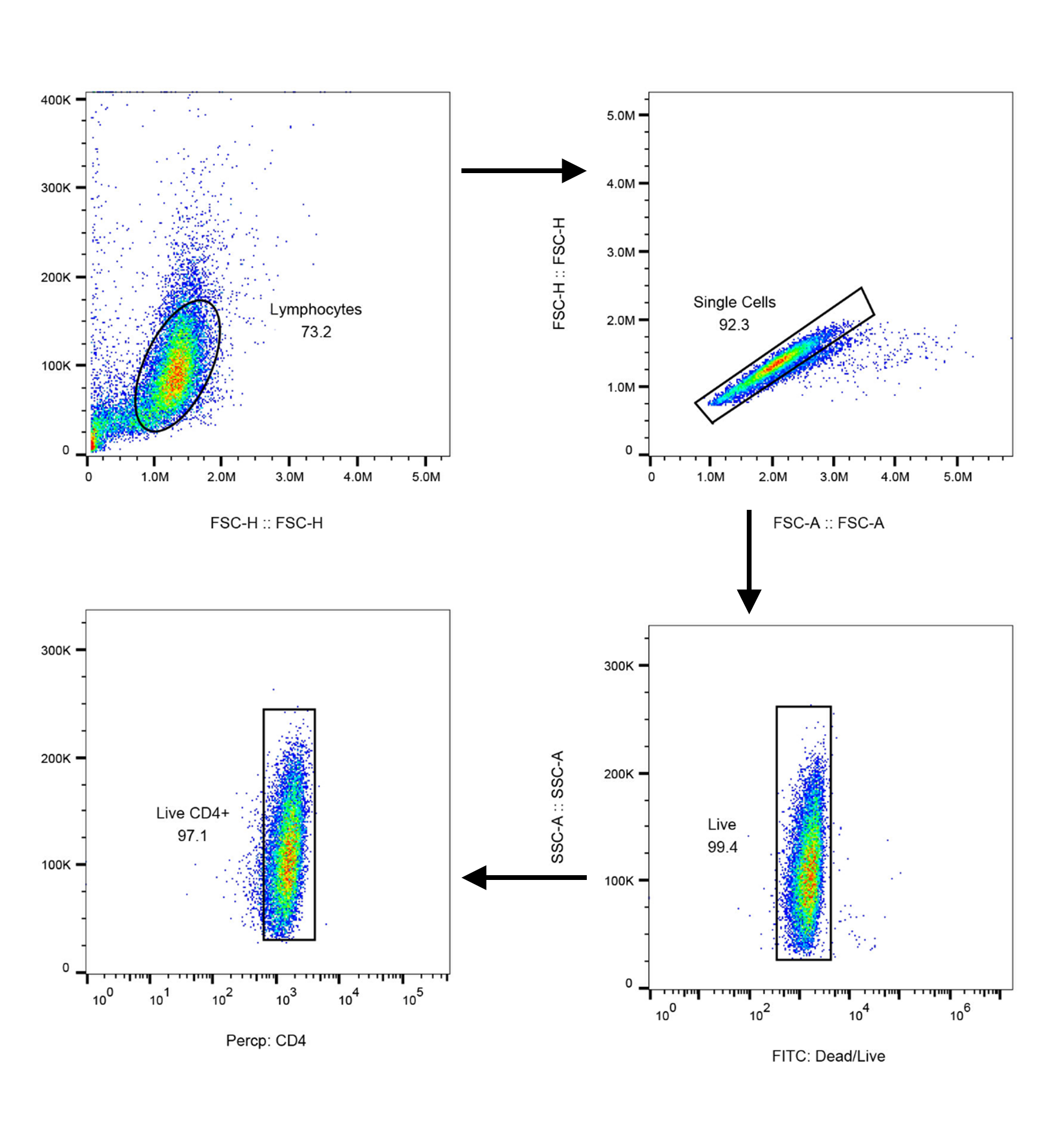

Supplement: Supplementary file 1 — Additional file 1: Figure S1. The purity of the isolated CD4+ cells was assessed by flow cytometry, and the percentage of live CD4+ cells achieved 97.1%. [file 13287_2023_3505_MOESM1_ESM.tif]

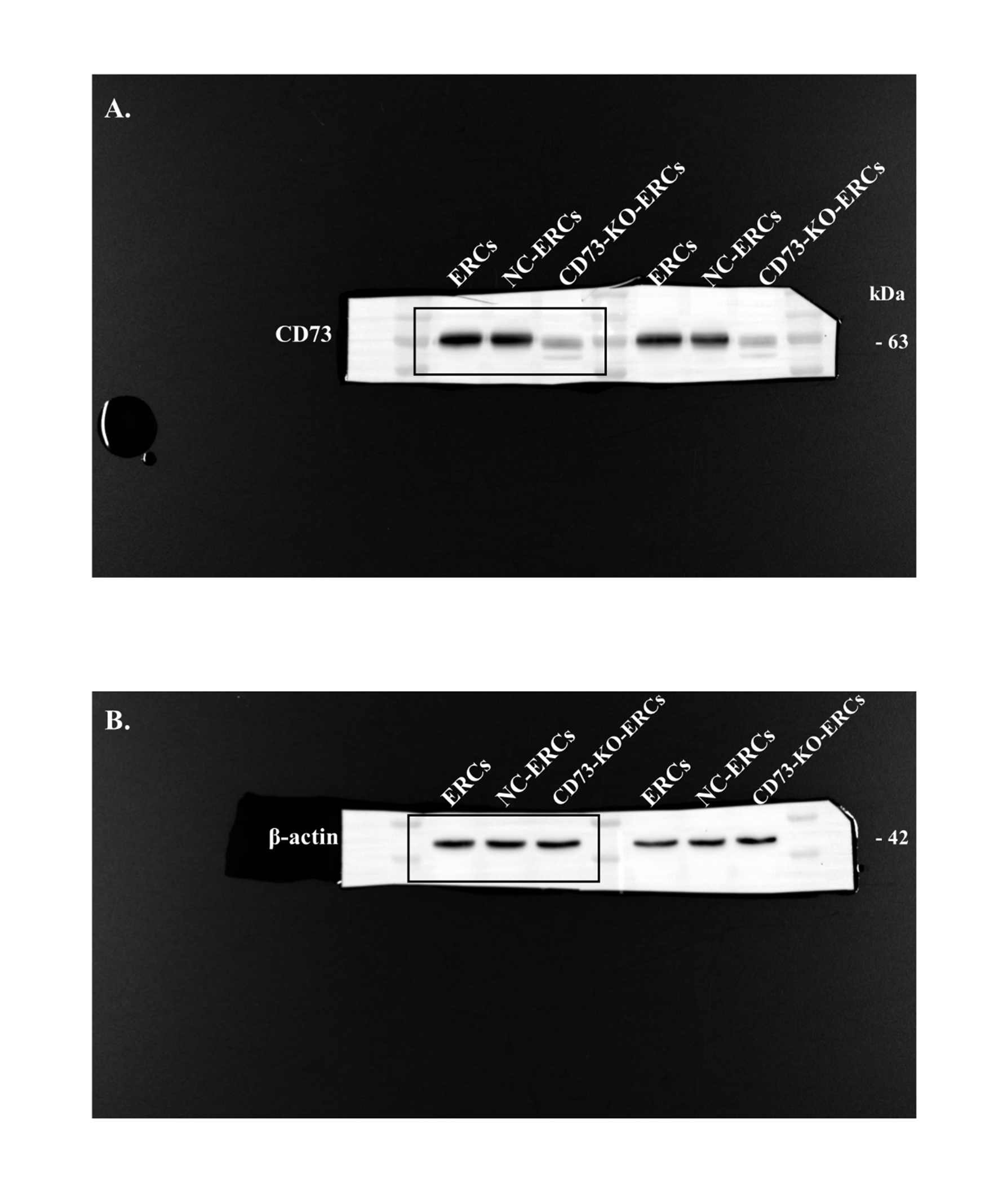

Supplement: Supplementary file 2 — Additional file 2: Figure S2. The Full-length merge blots of CD73 and β-actin in immunoblotting (protein). The shiny images used in the manuscript corresponded to the part marked here by the black frame. The lanes on the right above were duplicates. (A) The merge blots of C73. (B) The merge blots of β-actin. [file 13287_2023_3505_MOESM2_ESM.tif]

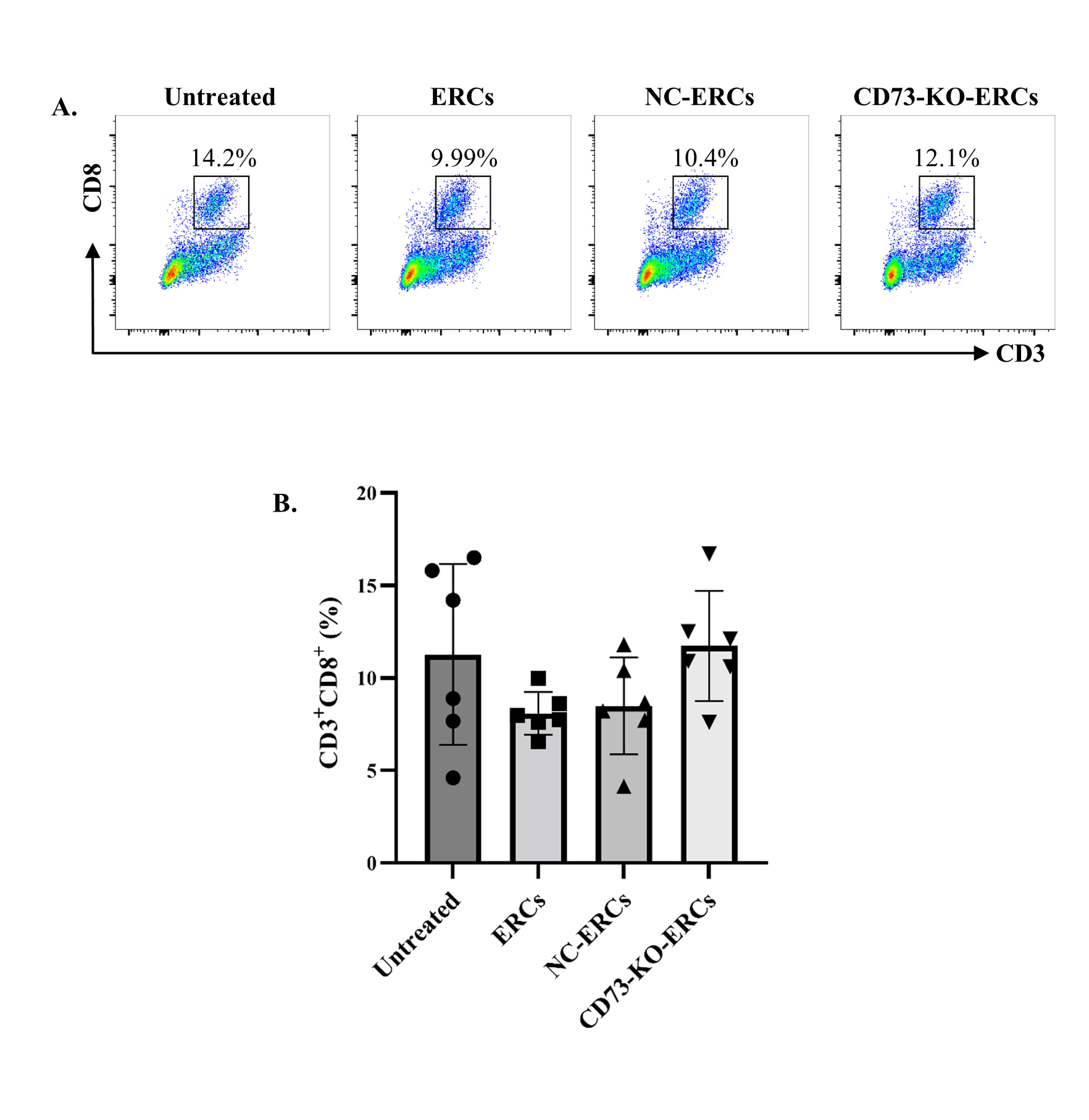

Supplement: Supplementary file 3 — Additional file 3: Figure S3. The liver infiltration of CD8+ T cells in different groups. (A) The representative pseudocolor plots were depicted. (B) show the statistical graph. One-way ANOVA was used for statistical analysis, no significance was found between those groups. Data in bar graphs represent mean ± SD. [file 13287_2023_3505_MOESM3_ESM.tif]

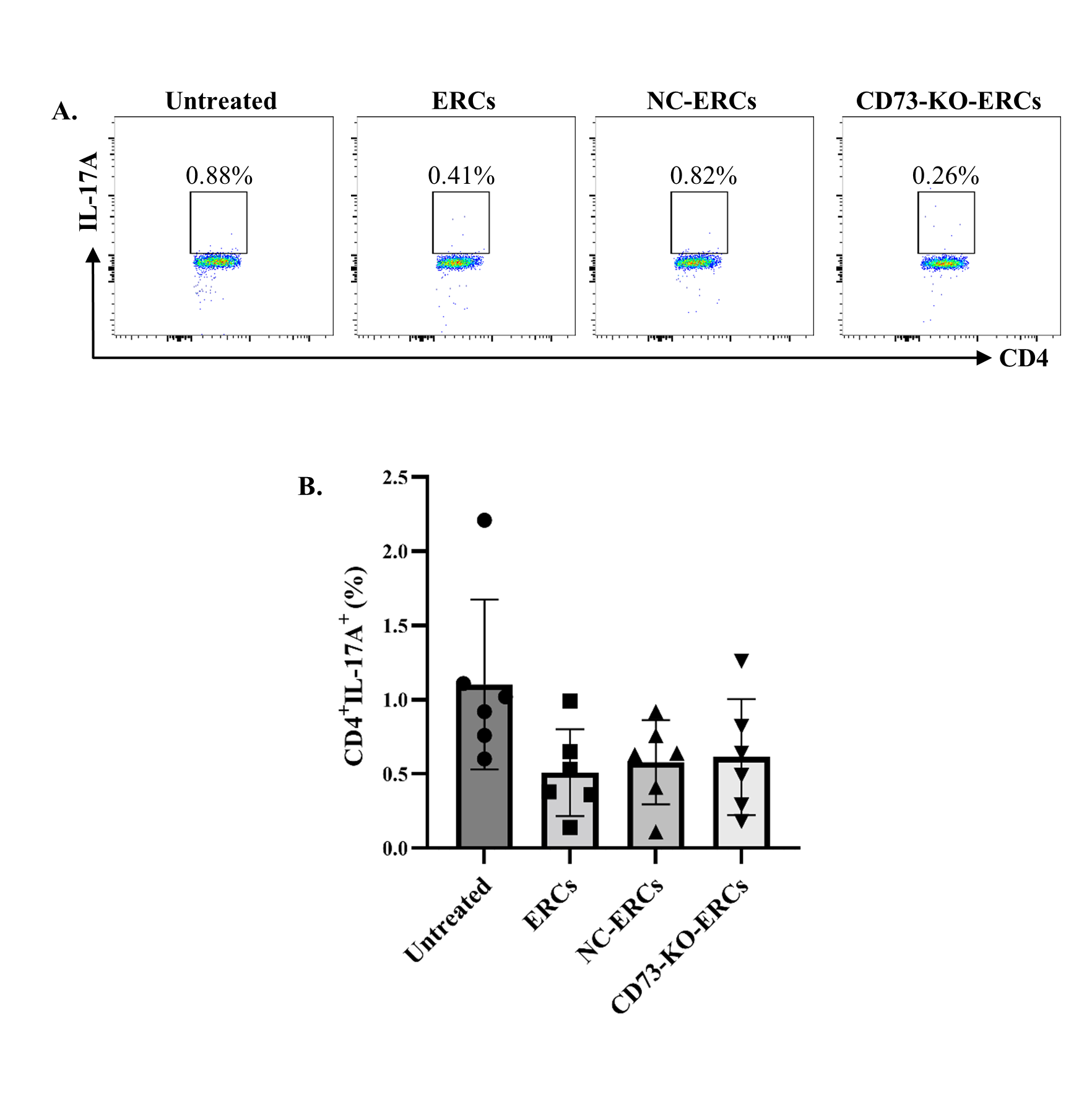

Supplement: Supplementary file 4 — Additional file 4: Figure S4. The liver infiltration of CD4+IL-17A+ Th17 cells in different groups. (A) The representative pseudocolor plots were depicted. (B) show the statistical graph. One-way ANOVA was used for statistical analysis, no significance was found between those groups. Data in bar graphs represent mean ± SD. [file 13287_2023_3505_MOESM4_ESM.tif]
